# Different antibody isotypes against tuberculosis: what we know and what we need to know

**DOI:** 10.3389/fimmu.2025.1682934

**Published:** 2025-10-22

**Authors:** Huoming Li, Hao Li

**Affiliations:** National Key Laboratory of Veterinary Public Health and Safety, College of Veterinary Medicine, China Agricultural University, Beijing, China

**Keywords:** *Mycobacterium tuberculosis*, tuberculosis, humoral immunity, antibody isotype, BCG

## Abstract

Humoral Immunity plays an important role during *Mycobacterium tuberculosis*(*Mtb*) infection. In mouse models, polyvalent and monoclonal antibodies targeting *Mtb* provided some protection against tuberculosis (TB). The five human antibody isotypes (IgG, IgM, IgA, IgE, and IgD) mediate an array of functional activities against bacterial infections, including neutralization, antibody-dependent cellular cytotoxicity (ADCC), phagocytosis, and complement activation. Different antibody isotypes have functions through different protective mechanisms based on the biological structures and pathways involved. In this review, we summarize the research progress on the different isotypes of antibodies against TB, and discuss the antibody-based strategies against tuberculosis, the potentiality of antibodies in TB diagnosis, and suggest further research directions, including investigating the mechanisms of different isotypes of antibody-mediated protection against TB, identifying correlates of immunity, and novel vaccines development.

## Introduction

Tuberculosis (TB) is still a leading cause of death from a single infectious disease. There were about 10.8 million cases of active TB globally, with 1.25 million deaths in 2023 (1). Currently, the only established TB preventive vaccine, *Bacillus Calmettee-Guérin* (BCG), because of its variable efficacy in adults, is not good enough to control TB despite its widespread use. Therefore, effective TB drugs and vaccines are the most urgent needs for TB control and treatment.

The mammalian immune system has innate and adaptive components including macrophages, dendritic cells, T cells, and B cells etc., which can cooperate to protect the host against bacterial infections (2, 3). In humans, the adaptive immune responses, including cellular and humoral immunity to *Mtb*, play an important role in the outcome of *Mtb* infection and disease progression (3–5). After Mtb enters the body, T cells and B cells can collaborate to control infection and direct the adaptive immune response. B cells have the functions to present *Mtb* antigens to activate T helper cells, which in turn stimulate B cells to produce antibodies and become memory B cells (6–8). Moreover, both cell types can produce cytokines, which can help regulate the immune responses to TB (6–8). Therefore, understanding the B-cell responses and protective roles of different antibody types in TB challenges the long-held dogma that cellular immunity alone is sufficient to fight Mtb infections.

In the late nineteenth century, it was thought that the animal’s serum therapy was effective in treating tuberculosis patients, but later the antibody effects were considered insignificant because of the inconsistent trial results (9, 10). There is now accumulated evidence that antibodies contribute to the prevention of *M. tuberculosis* infection and the progression of tuberculosis (11–19). However, evidence from passive monoclonal antibody (mAb) studies does not necessarily reflect the protective humoral immunity developed during a natural *Mtb* infection. Blocking of Th2 cytokines IL-4 can enhance host resistance and passive IgA protection against tuberculosis (20, 21). Understanding the antibodies’ roles, the targeting antigens, and the protective mechanisms will help us develop more effective diagnostics and novel TB vaccines in a more rational way (22). In this review, we summarize the research progress about antibody subclasses, various antibody effector functions, the antibodies in TB diagnosis, and raise important and potential scientific questions for TB antibody research in future studies, which can be a reference and help in the design and development of novel vaccines and therapeutics.

## Antibody isotypes and subclasses

**Figure 1:**
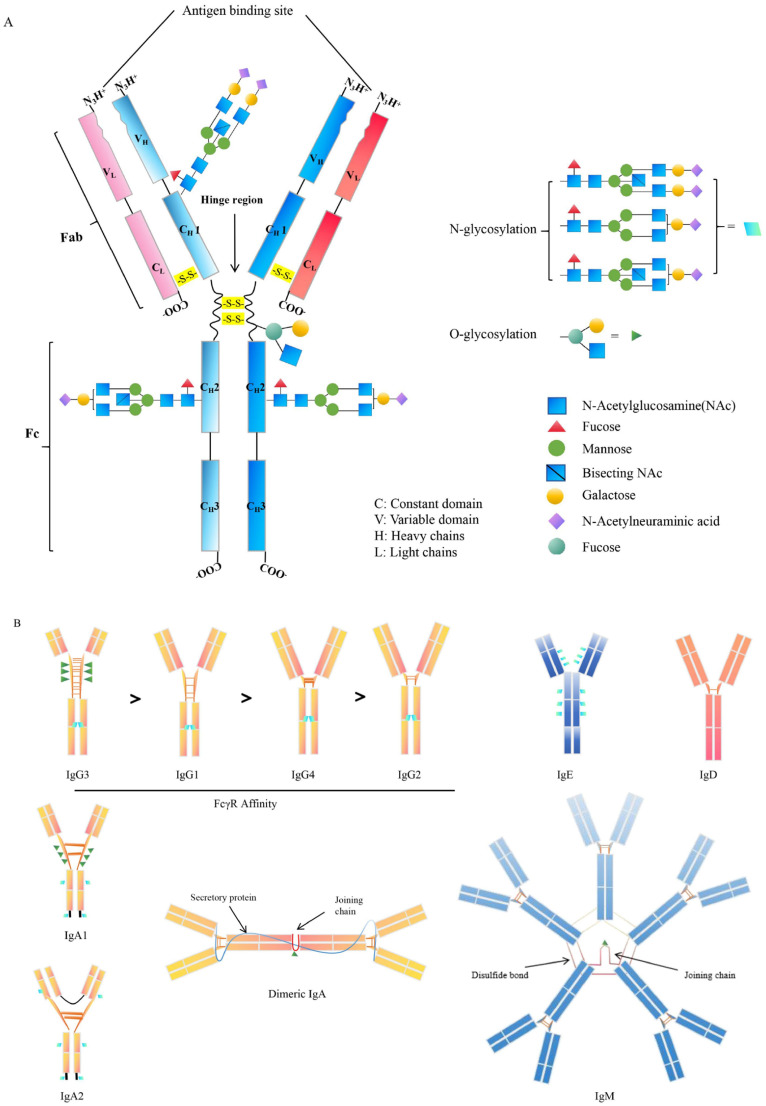
Structure of antibodies’ isotypes and subclasses. **(A)**. The general structure of antibody. The variable glycosylated chains are distributed in different antibody fractions such as the Fab and Fc domain. **(B)**. The structure and characteristics of antibody isotypes. The IgG is classed into 4 subclasses, including IgG1, IgG2, IgG3, and IgG4, according to the variations in heavy and light chain disulfide bonds. IgG subclass identified by FcγR affinity. In particular, IgG and IgE are only present as monomers, while the IgA exists either as monomers, such as IgA1 and IgA2 or as dimers. Additionally, IgM is solely presented as multimers, primarily as pentameric structures. N-glycosylation sites: ; O-glycosylation sites.

The antibody is a Y-shaped or T-shaped heterodimeric protein consisting of two heavy chains and two light chains, which are secreted by the plasma B cells (23). B cell receptor (BCR) -ligand interactions play a critical role in regulating B cell behavior, and primary B cell development and survival (24). The antibodies are further classified into five isotypes, named IgM, IgA, IgG, IgE, and IgD, depending on the unique constant region of the antibody (25) (**Figures 1A, B**). Major antibody isotypes display distinct serum abundances and half-lives: IgG (70–80% of total serum) has a ~21-day half-life; IgA (~15%) shows a short monomeric half-life (~1 day) but a longer secretory dimer half-life (~5–6 days); IgM (~10%) has a ~5-day half-life; IgD (<0.5%) and IgE (<0.01%) have short half-lives (~2–3 days and ~2 days, respectively) (26–28). Different quantities of antibodies and persistence can also affect immunological effects. For example, the extended half-life of IgG, mediated by FcRn recycling, is why this class is responsible for long-term immunity following vaccination or infection (26–28). The differences in antibodies’ constant regions could be reflected in the fact that each antibody isotype plays different roles throughout the infection process (29, 30). The antibodies have considerable diversity in the location and number of the conserved N-linked glycosylation sites that are located at the Fc as well as Fab regions. For IgG, it bears a single N-linked glycosylation site at asparagine 297 (N297) of each heavy chain and has shown importance in antibodies’ functions. The antibodies’ hinge region can contain N- and O-linked glycans (31, 32) (**Figure 1A**). IgG is the most important isotype in blood and extracellular fluids, while IgA is mainly secreted by plasma cells within mucosal membranes lining the intestines, airways, and reproductive tracts. IgG has a higher efficiency in regulating macrophage phagocytosis compared to IgA because of its functional location and easy access to T helper cells and molecules (33). IgA is the primary antibody protecting the mucosal surface, excelling at defending against invaders that can penetrate the mucosal barrier, with a unique tail structure that could resist acids and enzymes (34). IgE levels are very low in the blood and primarily bound to mast cell receptors located in the skin and submucosa. Antigen-binding IgE has induced mast cells to release chemicals to control pathogenic spread (35). Most antibodies are diffusely distributed throughout the body from the site of synthesis, while secretory IgA needs to be transported to the apical surface through the polymeric immunoglobulin receptor (pIgR) (36). Different isotypes of antibodies have been developed to work in various bodily regions. These antibody isotypes exhibit variable characteristics at different body locations to counteract pathogenic infections. Specifically, each antibody isotype has a specific structure that affects its function. For example, IgM, which exists as a pentamer, enhances antibody-antigen avidity in the form of multi-site binding and the ability to bind complement (37, 38). In the case of IgD, the heavy chain glycosylation leads to the formation of a T-shaped structure that increases the flexibility of the hinge region, which could well facilitate the epitope binding of the antigens and the synergistic action of IgM in the early stages of pathogenic infection (32, 39). IgD and IgG share the same basic structure but have a longer hinge region that is easily hydrolyzed by proteases (40). The other isotypes of antibodies, IgA and IgE, are smaller and can diffuse easily out of the blood into the tissues (40).

In addition to this, each antibody is further subdivided into several different subclasses based on the amino acid composition of the hinge region and the number and location of disulfide bonds (41). In mammals, there is only one type of IgM except for cattle (*Bos taurus*), which has two Igμ genes on the same chromosome and expresses two subclasses of IgM, IgM1 and IgM2 (42). Human IgG can be classified as IgG1, IgG2, IgG3, and IgG4, while mouse IgG is classified as IgG1, IgG2a/c, IgG2b, and IgG3. IgG1 is also the most promising subclass for tumor immunotherapy, and because human IgG1 is also able to bind effectively to the murine Fcγ receptor, significant effects can be observed in an *in vivo* mouse model (11, 43, 44) (**Figure 1**). IgG2 is mainly used to neutralize pathogen toxins or block the binding of receptor ligands, and its measured complement-dependent cytotoxicity (CDC) and antibody-dependent cell-mediated cytotoxicity (ADCC) effects have been shown to be very weak (45) (**Table 1**). IgG3 has the strongest binding capacity to FcγRs, triggers ADCC and antibody-dependent cell-mediated phagocytosis (ADCP), and has a stronger CDC effect than IgG1 (43–45) (**Table 1**). The hinge region of the IgG4 molecule is short, and its binding to FcγRs was weak (43, 55) (**Figure 1**). In humans, there are two subclasses of IgA, IgA1 and IgA2. Circulating monomeric immunoglobulin A (mIgA) and dimeric secretory IgA (sIgA) are two structures of IgA antibodies. Monomeric IgA is mostly IgA1 in the bloodstream and has functions as systemic immunity, while dimeric sIgA is the principal antibody of external secretions and the mucosal immune system (42). The differences between IgA1 and IgA2 are mainly in the structure of their hinge region and the number of glycosylation sites (57). In serum, the ratio of IgA1 and IgA2 is 9:1, whereas in mucosal tissues, IgA1 and IgA2 are evenly distributed. By contrast, mice have only one IgA isotype and lack a functional homolog to FcαRI, which is different from human beings (57) (**Table 1**). The studies indicate that IgA effector functions depend on subclass and glycosylation, and the balance of subclass distribution and IgA1/IgA2 ratio is associated with host immune responses and disease progression (58), and the mechanisms of these need to be further investigated.

**Table 1:** Different classes and subclasses of antibodies and their Fc fractions’ functions.

Human antibody isotype	Human antibody subclass	Mouse antibody subclass	Fc fractions’ functions	References
IgM	IgM	IgM	IgM has only one isotype in mammals, except for cattle (*Bos taurus*), which express two subclasses of IgM, IgM1 and IgM2. Regulate primary infections. Activate complement systems in humans and mice. IgM from mice can eliminate pathogens, especially in the primary infection period.	(46–48)
IgD	IgD	IgD	The function is similar to humans compared to mice, including regulating B cells’ maturity and selection.	(49, 50)
IgG	IgG1	IgG1	A main antibody subclass in blood; it binds effectively to the murine Fcγ receptor. IL-10 could act as a switch factor for IgG1 and IgG3. Activated efficiently complements the cascade reaction in humans contrast to the mouse.	(11, 43, 44, 51–53)
	IgG2	IgG2a	Neutralize pathogens’ toxins or block the binding of receptor ligands; Complement-dependent cytotoxicity (CDC) and antibody-dependent cell-mediated cytotoxicity (ADCC) effects.	(45, 46, 54)
		IgG2b	IFN-γ can induce the production of IgG2. Lipoarabinomannan (LAM) from Mtb induces IgG2 production. In mice, IgG2a has a stronger complement-activating ability compared to IgG2b, which could regulate immune reactions by FcγRI, FcγRIII, and FcγRIV.	
	IgG3	IgG3	Strongest binding to FcγRs. Trigger ADCC, ADCP, and the strongest CDC. In mice, IgG3 has a long half-life and the strongest binding capability to antigens.	(43–45, 52, 53)
	IgG4	–	Binding to FcγRs was weak. It plays an essential role in anti-inflammation and hypersensitive stimuli. IL-4 and IL-3 can induce the production of IgG4.	(43, 55, 56)
IgA	IgA1	IgA	In serum, the ratio of IgA1 and IgA2 is 9:1, whereas in mucosal tissues, IgA1 and IgA2 are evenly distributed.	(57, 58)
	IgA2		The mice have only one IgA isotype and lack a functional homolog to FcαRI, which is different from humans.	
IgE	IgE	IgE	The main function of the isotype was regulating hypersensitive reaction and anti-parasite immune response, which was similar to mice compared to humans.	(59, 60)

Different cytokine stimulation *in vitro* can induce B cells to secrete different antibody subclasses: IFN-γ can induce the production of IgG2 (61), while IL-4 and IL-3 can induce the production of IgG4 (56), and IL-10 can act as a switch factor for IgG1 and IgG3 (51) (**Table 1**). Therefore, this area regarding the mechanisms of antibody subclass protection deserves to be explored in depth in the future.

## The roles of antibody isotypes in TB prevention and therapies

**Figure 2:**
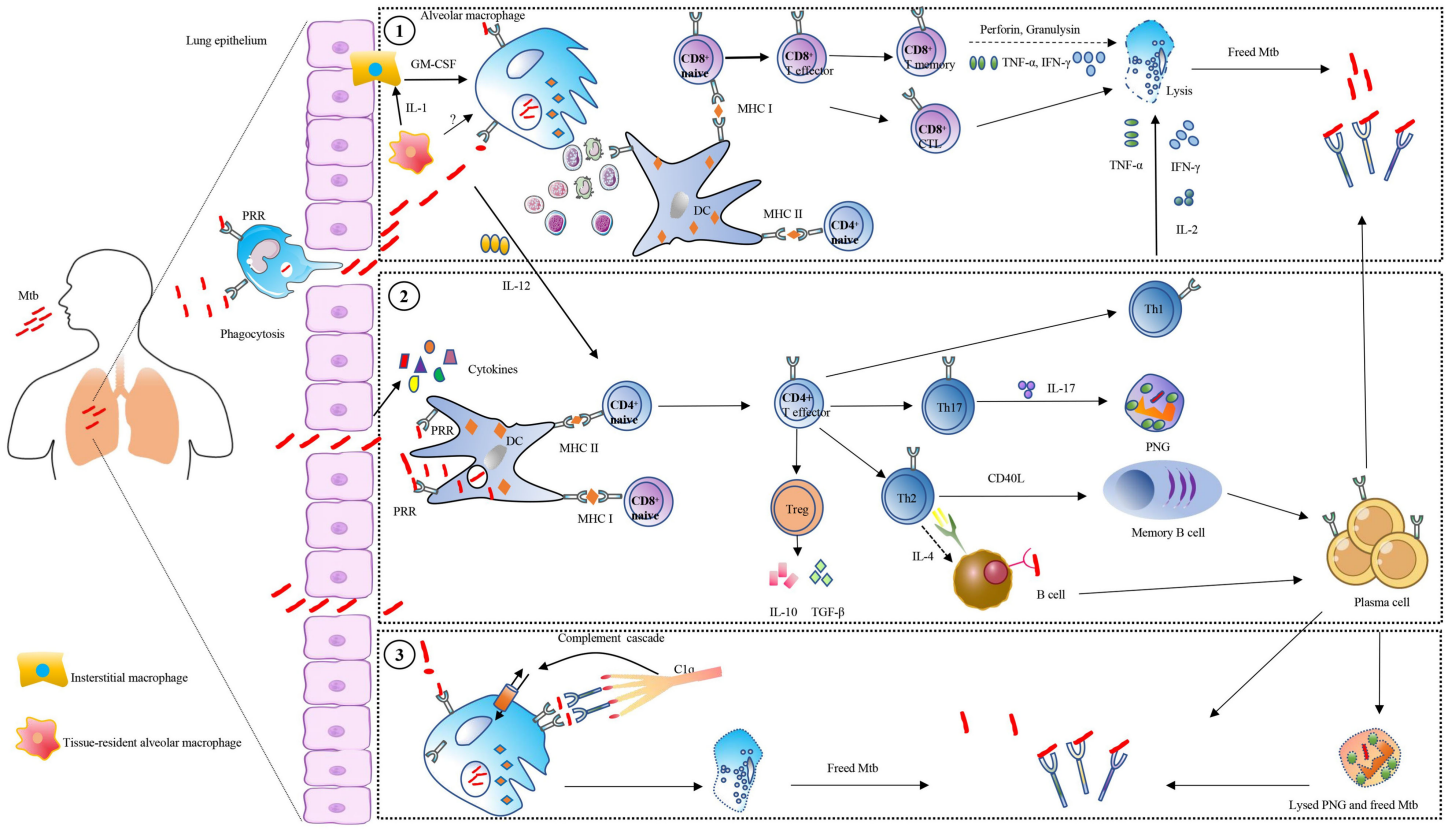
Overview of antibodies’ roles in tuberculosis. Control of Mycobacterium tuberculosis (*Mtb*) is the result of multiple immune cell interactions, such as T-cell, B-cell, macrophages, and dendritic cells (DC). Meanwhile, a variety of receptors and cytokines also mediated the process. The *Mtb* is internalized into macrophage and DC *via* pattern recognition receptors (PRR), and survives inside the phagosomal compartment, after which the apoptosis bodies carrying mycobacterial peptides are released. Several ways are employed by host cells to control *Mtb*: (1), These vesicles are acquired by DC, and the mycobacterial peptides are loaded on the MHC class I or MHC class II, which is presented to CD4+ T cells or CD8+ T cells. The CD8 T cells combined with MHC class I are activated, and act as cytolytic T lymphocytes (CTL) secreting perforin and granulysin to lyse host cells and kill *Mtb*. Moreover, the CD4+ T-helper (Th) cells combined with MHC class II are activated and polarized into different subsets including Th1, Th2, Th17, and Treg. Among them, Th1 cells can produce IL-2, TNF-α, and IFN-γ for interacting with macrophages. Th2 cells produce IL-4 to mediate B cells. Regulate T cells (Treg) produce IL-10 or transforming growth factor β (TGF-β). Th17 cells produce IL-17, which can activate polymorphonuclear granulocytes (PNG). (2), The *Mtb* is directly taken up by DC and is loaded on the MHC class I or MHC class II, with serial cells mediating as described above. (3) The affinity of antibody Fc domain with complement component 1 (C1q) causes complement-dependent cytotoxicity (CDC), which regulates host cells to eradicate *Mtb*. The lysed cells release *Mtb* which is affected by antibodies during cell-to-cell transfer.

The aerosol containing *Mtb* enters the human lungs and is phagocytosed by the resident alveolar macrophages (62–64). The tissue-resident alveolar macrophage can secrete IL-1 to activate interstitial macrophages for producing GM-CSF to activate monocyte-derived macrophages, which can lead to lung tissue damage (62–64) (**Figure 2**). Macrophages recognize the bacteria *via* different surface receptors, including complement receptors, Fc receptors, mannose receptors, and DC-SIGN receptor (65, 66). The phagosome and lysosome fusion is a critical pathway for inhibiting *Mtb* growth inside macrophages, and the granulysin and perforins secreted by CD8+ T cells can work together to decrease the viability of macrophage intracellular *Mtb (* 67, 68). Humans with IFN-gamma receptor deficiency are susceptible to Mycobacterial infection (69, 70). CD4+ and CD8+ T cells are important mediators of protection against *Mtb* infection (71, 72) (**Figure 2**). *Mtb* is a kind of intracellular pathogen that can arouse the Th1 type immunity, comprising monocyte activation and T cell cytotoxicity. B cells and T cells can work together to prevent *Mtb* infection. T helper cells can stimulate B cells to become activated and produce high-affinity antibodies, become memory B cells, and help activate cytotoxic T cells to kill macrophages (8, 73, 74). In the host granulomas B cells and T follicular helper (T_FH_) -like cells are important for TB control, and *Mtb* antigen-specific B cells can direct T_FH_ -like cells into lymphoid follicles, which can help in mediating *Mtb* control (75). On the other hand, B cells can modulate T cell immune response by different mechanisms such as antigen presentation, antibodies, and cytokines production (6, 7) (**Figure 2**). In primates, the subclasses of antibodies are related to the effectors’ response, such as cytotoxicity, phagocytosis, and secretion of immune cytokines (17, 76, 77). The amount and persistence of antibodies also have an important role in *Mtb* infection. The low quantity of antibodies may not be enough to confer protection, while the high amount of antibodies may not give protection, which is called a prozone effect (78–80). Studies indicate that the constant regions with the variable regions of antibodies can confer specific protective effects against *Mtb* infection (16, 17, 81, 82). Different murine isotypes of antibodies, such as IgM, IgG1, IgG3, and IgA, are passively protective against *Mtb* infection (**Table 2**).

**Table 2:** Antibody isotype-mediated protection against mycobacterial infections.

Isotypes	Target antigens	Cells or animals Models	Organism/antigen Challenge route	Timing of the administration of mAbs	Biological effects	Refs
IgM	Lipoarabinomannan (LAM)	Human acute monocytic leukemia cell line THP-1	Mtb	Incubated cells with Mtb in the presence of IgM	Increase the phagocytosis of mycobacteria	(83)
IgG1	Arabinomannan (AM)	Mouse (BALB/c)	*Mtb* (intravenous)	Mixed mAbs with *Mtb* before infection	CFU reduction and prolonged survival	(84)
IgG1	Lipoarabinomannan (LAM)	Human epithelial cells A549	*Mtb*	Preincubated *Mtb* with mAbs before infection	Inhibited the mycobacterial load in cells	(85)
IgG1	PstS-1	Mouse (BALB/c)	*Mtb* (aerosol)	Administrated intraperitoneally mAb before infection of 5 h	Reduced lung bacterial burden	(11)
IgG2b	MPB83	Mouse (BALB/c)	*M. bovis* (intravenous)	The mAbs incubated with *M. bovis* before infection	Reduced lung pathology and prolonged survival	(86)
IgG2b	OmpA	Mouse (C57BL/c)	*M. bovis* (intranasal)	Administrated intraperitoneally mAb before infection of 5 h; four injections of antibodies at 1-week intervals for mice treatment	Reduced lung pathology and bacterial burden	(87)
IgG3	Arabinomannan (AM)	Mouse (BALB/c and C57BL/c)	*Mtb* (aerosol intratracheal)	*Mtb* incubated with mAb before infection	Enhance host survival and *Mtb* confinements in granulomas	(88)
IgG3	Heparin-binding haemagglutinin (HBHA)	Mouse (BALB/c)	BCG (intranasal)	BCG coated with the anti-HBHA mAb	Reduction of the spread of illness	(89)
IgA	Heparin-binding haemagglutinin (HBHA)	Human epithelial cells A549	*Mtb* (aerosol intratracheal)	Preincubated *Mtb* with mAbs before infection	Isotype-dependent inhibitory adhesion	(85)
IgA	16kDa α-crystallin (Acr)	Mouse (BALB/c)	*Mtb* (intravenous)	Inoculation mAb at 2 h before and again at 2 and 7 d after infection	Reducing lung bacteria burdens, and IFN-γ inoculation can help enhance the effects.	(90)
IgA	16kDa α-crystallin (Acr)	Mouse (BALB/c)	*Mtb* (aerosol or intranasal)	mAb were inoculated at various dosing times pre- and post-challenge with *Mtb*	CFU reduction in early-stage infections	(91)
IgA1	16kDa α-crystallin (Acr)	Mouse (CD89tg)	*Mtb* (intranasal)	mAb mixed with IFN-γ was administered 2 h before infection	Reduced lung pathology and bacterial burden	(92)
IgA	LpqH	Mouse (BALB/c)	*Mtb* (aerosol)	Administrated intraperitoneally mAb before infection of 5 h	Reduced lung bacterial burden	(14)

### IgM

**Figure 3:**
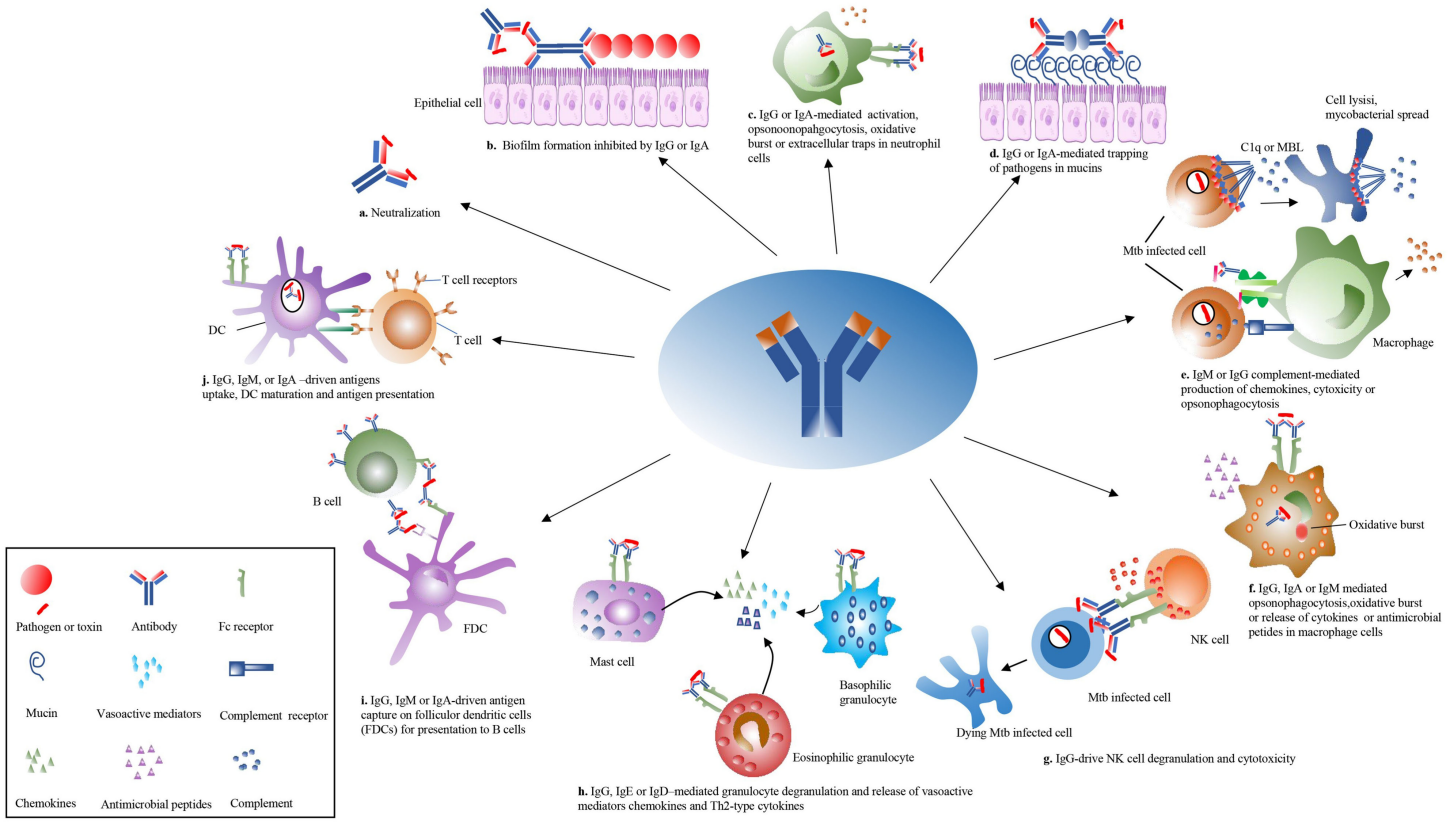
Overview of the antibodies’ effector functions. The antibody can play pleiotropic effectors functions during *Mtb* infection. **(a)** The antibody is directly neutralized with *Mtb*. **(b)** The antibody isotypes, IgG and IgA, inhibited biofilm formation. **(c)** The IgG and IgA activate neutrophil opsonophagocytosis, oxidative bursts, and extracellular traps. **(d)** The antibody isotypes, IgG and IgA, mediated the trapping of *Mtb* in mucins. **(e)** The IgM and IgG activate complement cascade to induce cell lysis through the membrane attack complex or drive *Mtb* clearance. **(f)** The IgG, IgM, and IgA mediate opsonophagocytosis, oxidative burst, or release of cytokines in macrophage cells. **(g)** The IgG isotypes drive natural killer (NK) cell degranulation to kill infected cells. **(h)** The IgG, IgE, and IgD mediate the degranulation of mast cells, eosinophilic granulocytes, and basophilic granulocytes to release vasoactive substances and cytokines in allergens or parasitic infections. **(i)** The follicular dendritic cells (FDCs) present mycobacterial antigen to B cells. **(j)** The IgG, IgM, and IgA mediate DC to enhance antigen uptake, processing, and presentation toward T cells. MBL, mannose-binding lectin.

IgM is expressed on the cell surface at the beginning of B-cell establishment in the bone marrow and accompanies the entire process of B-cell maturation. IgM is evolutionarily conserved and can specifically bind antigens in the absence of prior immunization (93, 94). IgM is the primary class of antibody produced early in host infection and provides a rapid antibody response. The protective roles of IgM against a wide range of pathogens, including intracellular bacteria such as *Mtb* and *Ehrlichia muris* (*E. muris*), have been demonstrated (95–100). IgM plays an indispensable role in the development of an optimal germinal center (GC) reaction, which is a prerequisite for the establishment of effective humoral immunity for chronic TB control. The variation of specific Ig classes may potentially affect TB disease progression (101). The immunodeficient mice, lacking IgM secretion, exhibit significant susceptibility to TB, indicating the protective role of IgM in TB progression (99, 102) Intravenous administration of the BCG vaccine can prevent *Mtb* infection in a rhesus monkey model (100), and the existence of *Mtb*-specific IgM in bronchoalveolar lavage fluids (BALF) of the BCG-vaccinated monkeys implies that IgM can have protection against TB in an early phase of *Mtb*-host interaction (98, 100). The above results initiate a new era in the study of IgM against TB and provide a new direction for the study of the mechanisms of humoral immunity in fighting against intracellular mycobacterial infection. However, early IgM antibodies do not undergo somatic hypermutation and therefore produce IgM with low affinity (33). Fortunately, IgM can form pentamers, which spontaneously bind multivalent antigenic molecules, such as bacterial capsule polysaccharides (103). Therefore, the deficiency of IgM monomer avidity is compensated by this multipoint binding ability (104). The rapid production and efficient activation of IgM can be very effective in controlling bacterial infections, which would have serious consequences if the pathogenic infection is not controlled as soon as possible. Specific long-lived IgM plasma cells can indeed demonstrate somatic mutations, produce IgM antibodies, and contribute to long-term protection (105). IgM can function as an antimicrobial activity by modulating multiple immune processes, including opsonization, dendritic cell functions, T cell immunity, and humoral responses (103, 106). During the active phase of TB, the IgM against different *Mtb* antigens is induced within one month of infection (107). In immunized rhesus monkeys, IgM titers are the strongest marker of reduced bacterial load, and intravenous BCG administration to rhesus monkeys elicited near complete immune protection against TB (98). The IgM exhibits very potent anti-bacterial and viral activity by fixing complement and mediating protection (108). Benefiting from its pentameric and hexameric structure, IgM has a high affinity to the complement component C1q and is therefore more likely than IgG to utilize the complement system to accomplish complement-dependent cytotoxic (CDC) processes (109) (**Figure 3**). BCG can bind to C1q in the presence of IgM in serum samples from BCG-vaccinated people (110). Mild tuberculosis meningitis (TBM) is associated with overall higher IgM titers to *Mtb* antigens in the CSF and is characterized by an enrichment of *Mtb*-specific antibodies that can activate complement and drive phagocytosis by monocytes and neutrophils (111). The mAb anti-LAM IgM A194 can inhibit *Mtb* growth in human whole blood but not in macrophages, which suggests that other factors, including complement, may be required for the restrictive effects (100). Mouse mAb anti-LAM IgM TMDU3 can bind C1q and iC3b, activate the classical complement pathway and enhance mycobacteria phagocytosis and promote the fusion of phagosome and lysosome in a CD11b-dependent manner (83). However, the detailed molecular mechanism by which IgM mediates the complement system against TB remains poorly characterized and warrants further investigation in future studies.

### IgD

In general, the IgM and IgD are co-expressed on the mature B cell surface before antigenic stimulation, and IgD may play a crucial role in regulating B cell maturation (25). IgD isotypes are not presented on the surface of primary B cells but on the mature B cells, and are mainly expressed in the upper respiratory tract, where they bind well to basophils and innate immune cells to promote bactericidal activity through unknown receptors (25). Structurally, IgD has the increasing flexibility of the hinge region, which greatly augments antigen-antibody binding. In addition, IgD also synergizes with IgM to have protection at the early stages of pathogenic infections to exert anti-infective effects (112). One study indicates that older TB patients have higher total IgD levels (113).

### IgG

Monoclonal antibodies of the IgG isotype have been utilized for passive therapy, since IgG is the most abundant antibody isotype in serum with a long half-life. The increasing evidence suggests antibodies play a protective role in mouse infection models, including BALB/c and C57BL/6 (13, 17, 114). Intravenous administration of the high dose of total human immunoglobulin IgG (hdIVIg) to mice resulted in significantly reducing the organs’ bacterial loads in *Mtb*-infected BALB/c and C57BL/6 mice models (15, 115). The hdIVIg may have protective effects, maybe because it can modify the critical cells’ responses against TB, such as dendritic cells and T cells (115). In another research, the human IgG was administered into mice *via* the intranasal route, and the results showed a remarkable decrease in pulmonary bacterial load in mice (116), in this study, the gamma-globulin’s protection was abolished after incubation with *Mtb*, which suggests a potential role of *Mtb*-specific antibodies against TB (116). The di-glycosylated glycan structures found on the Fc region of IgG can differentiate tuberculosis infection (TBI) and active tuberculosis (ATB), as well as discriminate treated ATB from ATB (27). Moreover, the TB-specific IgG4 antibodies are evaluated in ATB but diminished after TB treatment (27). The total Ig isolated from highly exposed healthcare workers was injected into the mice 5 hours before aerosol infection and can offer protection in the lungs’ CFU reduction (13). The abundance of IgG3 from protective donors in this study was higher than that from a nonprotective donor when testing for decay kinetics in the mouse model in this study (13). The functions of antibody subclasses could be affected by hinge region length and disulfide bonding, and longer, more flexible structures implied an increased binding efficiency for antigens, complements, and Fc receptors (117, 118) (**Figure 3**). Different subclasses may play different regulatory functions at different stages of TB development (119). The IgG1 antibody subclass against TB could increase the TNF-α release, responsible for disease localization and granuloma formation at the early stages of infection (120, 121). The IgG1 monoclonal antibodies directed against LAM named SMITB14 can increase the survival rate and reduce bacterial load and weight loss in BALB/c mice (84). The human IgG1 P1AM25 targeting oligosaccharide (OS) motifs of AM can enhance *Mtb* phagocytosis by macrophages, reduce intracellular growth in an FcγR-dependent manner, and have protective effects in passive transfer with *Mtb*–infected FcγR-humanized mice (114). However, P1AM25 in murine IgG2a but not IgG1 can give protection against *Mtb* infection in C57BL/6 mice (114). Another study indicates that the IgG1 of monoclonal anti-LAM increased the mycobacterial load in A549 cells (85). The presence of only neonatal receptors in the A549 cells, lacking conventional FcγR and FcαR receptors expressed on the cell surface, may be the main reason for the differences (85). Antibodies targeting polysaccharide LAM and protein antigens in TB patients were predominantly of IgG regardless of the patient’s clinical status (122). In murine models of *C. neoformans* infection, the switch from IgG3 to IgG1 increases antibody protective efficacy against *C. neoformans* infection (123). The mouse IgG2b monoclonal antibodies targeting OmpA and MPB83 have shown a significant reduction in the bacterial burdens in mice infected with *M. bovis* (86, 87). In TB patients, different antibody isotypes are found to target lipoarabinomannan (LAM), which is a mycobacterial cell wall glycolipid component (54, 122). A significant switch from anti-LAM antibody subclass IgG1 to IgG2 was observed from tuberculoid toward lepromatous forms, despite a constant total amount of antibodies, suggesting that this conversion may be associated with changes in antibody protective effects (122).

### IgA

In the early stages of *Mtb* infection, IgA antibodies secreted from the mucosal surfaces of the respiratory tracts play a key role in the anti-infection process (**Figure 3**). The anti-HBHA IgA monoclonal antibodies were shown to inhibit bacterial uptake in lung epithelial cells (85). The use of arabinomannan reactive monoclonal antibodies to opsonize *Mtb* demonstrates that IgG1 on THP1 monocytes has no significant difference in bacterial counts compared with the isotype control, whereas the IgA1 isotype does (85). A monoclonal antibody targeted *Mtb* surface lipoprotein LpqH from a highly exposed but uninfected healthcare worker, which was identified to be of IgA isotype in its natural form, was shown to have protection against tuberculosis (14).

Antigen specificity influences protection, since a mouse IgA monoclonal antibody binding to the Acr antigen named TBA61 was found to be far more protective than an antibody TBA84 against the PstS1 antigen (91). Mouse IgA monoclonal antibodies, regardless of their specificity, can inhibit the proliferation of mouse macrophage cell lines. The anti-proliferative activity is manifested by IgA binding to J774.1 cells, stimulating tumor necrosis factor (TNF)-alpha production and inducing apoptosis, but not by mouse monoclonal IgG and IgM (124). The TNF-α is essential for granuloma formation and macrophage recruitment at the early stages of *Mtb* infection; however, too much TNF-α can lead to the necrosis of the infected macrophage cells and help the mycobacteria release into the blood (125, 126). In addition, adding IFN-γ before macrophages infected with IgA-opsonized *Mtb* can increase nitric oxide and TNF-α production, and decrease the bacterial counts in macrophages (90). When Balb/c mice were inoculated with mouse IFNγ and an anti-Acr IgA TBA61 mAb *via* the intranasal route (i.n.), a synergistic protective effect on reducing bacterial burdens can be found for the lungs harvested 3 and 4 weeks after H37Rv aerosol infection, while neither component alone was protective (90, 127) (**Table 2**). Moreover, when co-inoculation of anti-Acr IgA TBA61 mAb and mouse IFNγ *via* i.n. route in Balb/c mice with IL-4 depletion by a neutralizing anti-IL4 mAb, a significant reduction of bacterial burdens can be observed compared with IgA and IFNγ treatment in wild-type mice after H37Rv infection (21) (**Table 2**). The studies indicate that the IFNγ and IL4-mediated macrophage functions are involved in the process which anti-*Mtb* IgA mAb inhibit *Mtb* growth (21, 90, 127).

### IgE

Specific IgE has been reported in TB patients, suggesting that IgE may play an important role during TB disease progression (56, 128, 129). In TB patients, IgE levels are significantly elevated, and a decrease in IgE levels after treatment is observed. In addition, total IgE levels are significantly higher in TB patients with intestinal helminths and human immunodeficiency virus (HIV) co-infection than in those with helminths or without co-infection (p< 0.05) (130). Specific IgE levels are elevated in both tuberculosis and leprosy patients, and the differences can be observed between TB patients and healthy controls (131). Therefore, changes in the level of total IgE are often ignored in TB diagnosis (132, 133). Conventionally, IgE is often used as a characteristic marker of allergic diseases, and the relationship regarding specific IgE and TB is still not very clear (133).

## The roles of antibody isotypes in TB diagnosis

The main component of immunologic diagnosis of TB is based on the detection of antibody responses to *Mtb* antigens. *Mtb* infection provokes a complex humoral immune response, with different antigens expressed at different stages of the infection (134, 135). Studies have shown that antigen or epitope-specific serology may help in diagnosis, assessment of prognosis, and monitoring of chemotherapy in TB patients (136–138). The antigens selected for antibody detection are important. A systems-level analysis of the antibody response to the entire *Mtb* proteome in TB patients indicates that *Mtb* immunoproteome is rich in membrane-associated and extracellular proteins, and the antibody responses to the same antigens varied among patients and are correlated with bacillary burden (139). Using serology for active TB patients finding could reduce the TB transmission, which can help treat the patients and limit the spread timely (140, 141). The previous studies have shown that the antigens such as 38-kDa antigen (PstS1), the 16-kDa antigen (Acr), and LAM are the immunodominant markers for ATB diagnosis (142–144). The smear-positive pulmonary tuberculosis patients have increased serum immunoglobulin titers against mycobacterial antigens; however, there are still 10% didn’t show any increase (145, 146). On the other hand, antibody-based assays have performed poorly when used to diagnose sputum smear-negative TB, and antibody responses can also be observed in past TB cases, which pose additional challenges for TB serodiagnosis (147).

As the most predominant antibody isotype in serum, IgG has been the focus of TB diagnosis. Serologically based detection of IgG levels of single or multiple antigens is by far the most common concept in TB diagnosis. 11 *Mtb* antigens are combined to detect IgG levels, and the results showed an astounding sensitivity of over 95% in sputum smear-positive samples (148). In 755 HIV-uninfected adults, the three-antigen model and the multi-antigen model have shown higher sensitivity compared with the single-antigen model (149). As such, IgG level against TB in the context of polyprotein fusion from TB antigens is used in diagnosis to discriminate TB patients. In terms of diagnostic sensitivity of up to 90%, it shows that six antigen fusion became an effective way to improve TB detection (150). The reason for using multiple antigens to detect TB was that each antigen is expressed at a different stage of infection, and the use of multiple antigens allowed for a more accurate diagnosis. Similarly, each antibody subtype and subclass significantly different at the stage of TB development, even when the total amount is constant. Therefore, the combined diagnosis of multiple isotypes becomes a possibility for efficient diagnosis. Moreover, the characteristics of the antibody are also a factor for an accurate diagnosis to distinguish the active TB and TBI. Some groups have revealed the presence of distinct glycosylation patterns in IgG Fc portion antibodies in active TB and TBI from South Africa, the USA, and Mexico, implying that the glycosylation could be a potential molecular target to differentiate between active TB and TBI (15, 27). Recent studies have shown that subclass IgG4 also serves as a new antibody signature for active TB, especially after significant changes in treated TB patients, indicating potential as a signature molecule for detection (27). FcγRI, an activating receptor of immune cells, can be significantly upregulated by IFN-γ and GM-CSF and binds to monomeric IgG1, IgG3, and IgG4 with a high affinity (151). Therefore, the FcγRI levels can be a marker that would help to improve the sensitivity of the detection to distinguish between the active and latent infection. Apart from that, the IgG1 and IgG3 are also major antibody subclasses for complement activation, with significantly higher levels of complement C1q in active TB patients compared to those with latent infection (52, 53). Therefore, the complement C1q expression related to active TB could be a potential marker to discriminate the TBI from active TB. The high-affinity antibody receptor FCγR1A, which principally binds the IgG1 and IgG3 subclasses, has been observed to be higher by analysis of whole blood transcription in active TB patients than in those with TBI, regardless of HIV status or ethnicity (152). Therefore, the FCγR1A expression level has the potential to be a biomarker for indicating acute tuberculosis in patients.

*Mtb* antigen-specific IgA antibodies could be used to develop accurate tests for TB diagnosis, the studies suggest that IgA targeting Acr could discriminate between clinical TB patients and healthy controls (153, 154). Moreover, the anti-16kDa IgA and anti-MPT64 IgA have been found suitable target molecules to discriminate the active TB and TBI, with >90% sensitivity in diagnosis (155). The role of IgM in TB diagnosis is not well understood. The previous studies indicate that the diagnosis of patients with TB by measuring the titer of IgM antibodies in serum alone showed low sensitivity (156, 157). However, the combination of IgG, IgM, and IgA antibody responses to protein antigens or polysaccharides like LAM can improve the sensitivity and specificity of active TB diagnosis (155, 158).The different mycobacterial species may have their unique characteristics of LAM structure. Rapid-growing nontuberculosis mycobacteria (NTM) such as *M. smegmatis* have uncapped ends or inositol phosphate caps (PILAMs), and slow-growing NTM such as *M. avium* are capped with mannopyranose residues, which leads to manLAM (159, 160). Therefore, it is important and possible to develop antibodies specifically targeting *Mtb* regions to enhance the specificity (161). Antigen variation in different lineages of *Mtb* should also be considered when the *Mtb*-specific antibodies are used for TB diagnosis. There is a 63bp deletion in *Mtb* lineage 4.2.2(L4.2.2) strains, which may affect the MPT64-based testing results in L4.2.2 isolates prevalent areas (162). Therefore, we should consider the changing levels of antibodies and also epitopes recognized by the diagnostic antibodies when conducting the TB diagnosis protocol design to effectively improve the accuracy and performance of the test.

## Conclusions and perspectives

Tuberculosis is a contagious respiratory disease due to *Mtb* infection and is one of the top 10 single pathogens in the world in terms of mortality (163, 164). The problem of antibiotic therapy for tuberculosis has led to the emergence of *Mtb* drug resistance. In recent years, passive therapy with antibodies has become increasingly popular for research as an alternative to antimicrobial therapeutic agents. The effectiveness of various antibodies relies mostly on isotypes, which allows them to efficiently adapt to the most appropriate mode of transport across the epithelium to the site of function. Additionally, crucial research tools include broadly and powerfully antibodies, which may be used to find protective epitopes that can be developed into functional vaccines by structure-based reverse vaccinology. Currently, more than 70 monoclonal antibodies have been approved for the treatment of various pathogenic bacterial infections (165). However, many hurdles remain in the field of anti-infective mAbs: finding optimal targets for a pathogen, understanding how the isotypes, Fc receptors (FcRs), and other structural regions mediate protection, and developing better pre-clinical and clinical trials to investigate the therapeutic potential of these antibodies (165).

Traditionally, it is believed that, as intracellular bacteria, the protective immune response against TB is mainly exerted by T cell-mediated cellular immunity, including CD4+ and CD8+ T cells (166). However, in recent years, new findings suggest that B cells also play an important role in the anti-TB process, but the exact mechanisms are not well understood (167). Currently, BCG is the most commonly used TB vaccine. Its role is preventing the onset of meningitis and disseminating TB in infants and children. According to the World Health Organization in 2017, 120 of the 158 countries that allow BCG immunization have 90% BCG immunization coverage (168).

However, the failure of BCG to protect against pulmonary disease in adults has limited its use in a larger range of populations. In addition, significantly different immuno-protective effects were observed with different immunization routes of BCG, with significant immuno-protective effects observed with mucosal immunization with BCG compared to subcutaneous immunization (169, 170) and in intravenously BCG-injected experimental monkeys (98)Changes in both cellular immunity-related factors and antibody levels have been observed in these studies, but their roles in host protection and their modes of action remain to be answered.

Previous studies have mostly focused on eliciting cell-mediated immune responses against TB (171–173). More and more studies have shown that the host can produce protective antibodies against TB, and an increasing number of *Mtb* antigens have been reported to have the ability to induce protective antibodies (11, 14, 17, 174). Therefore, the integration of humoral immunity into tuberculosis vaccine development could be a potentially effective strategy. Recent studies have shown that the subunit vaccines incorporating the antigens arousing cellular immunity, such as Ag85A, and humoral immunity, such as PstS1 or LpqH, can significantly enhance the protective efficacy against TB (22, 175, 176), which gives us suggestions that it is rational to design the TB vaccines based on both cellular immunity and humoral immunity.

An ideal target is a prerequisite for an antibody to have its functions. It is worth investigating whether specific antigens, their relative concentrations, and post-translational lipid and carbohydrate moieties influence the class or subclass of antibody production in future studies. *Mtb* has secreted proteins playing important roles in its virulence and immune evasion. The secreted virulence proteins are secreted by secretion systems and can modulate the host immune responses (177). Therefore, some secreted proteins are possible therapeutic targets for TB treatment. The development of antibodies targeting secreted systems and proteins is a rational strategy for TB control and treatment. The cell walls of *M.bovis* BCG resemble those of Gram-negative bacteria and are presumed to have a four-layer membrane structure, from inner to outer: the cytoplasmic membrane, the peptidoglycan-arabinomannan complex, the extracellular membrane, and the outermost pod membrane (178). The presence of 144 outer membrane proteins in *Mtb* was deduced using signal peptide prediction, transmembrane protein prediction, and β-strands amphiphilicity, but only MctB and OmpATb of *Mtb* and MspA of *M. smegmatis* have been fully identified (179, 180). Antibodies that recognize bacterial outer membrane proteins generate immune protection (87), suggesting the potential value of screening efforts for antibodies against outer membrane proteins (87, 165). Given that most antibodies recognizing surface proteins are currently uncharacterized, their anti-infective value awaits further elucidation.

The antibody-dependent enhancement of infectious disease should also be considered. The antibodies’ receptor FcγRI inactivation can impact nitric oxide production by neutrophils, antigen presentation, and antibody-dependent killing of pathogens by macrophages (181, 182). Moreover, the improved *Mtb* control in the lungs of *Fcgrt^−/−^* mice compared to the control mice was observed and associated with reduced neutrophil recruitment (181, 182). Neutrophil accumulation is associated with increased disease severity in human TB and in mouse models of TB (183–185). Moreover, one study indicates that rabbit anti-H37Rv sera can facilitate the multiplication of BCG in the spleens of mice (186). Antibodies’ prozone-like effects should also be considered when developing antibody-based treatment methods for TB (78–80). The Mtb ‘decoy’ constituents also need to be considered. Antigens such as PstS1 may exacerbate disease by inducing a Th2 response, while anti-Acr antibodies show potential for protection, but the Th2 stimulant components need to be removed (21, 143, 187). Although obstacles remain in identifying effective targets and understanding how monoclonal antibodies protect against different infections, progress in these areas is a positive indication that monoclonal antibodies will be more widely accepted in the future as a treatment for bacterial infections.

Above all, the different roles of antibodies’ isotypes in tuberculosis need to be further investigated. The discovery of protective antibodies against TB can contribute to TB prevention and treatment. At the same time, the antigens identified following the isolation of protective antibodies will help in the design and development of novel tuberculosis vaccines.
